# Associations of *HLA-DP* Variants with Hepatitis B Virus Infection in Southern and Northern Han Chinese Populations: A Multicenter Case-Control Study

**DOI:** 10.1371/journal.pone.0024221

**Published:** 2011-08-31

**Authors:** Jin Li, Daguo Yang, Yongwen He, Mengyi Wang, Zirong Wen, Lifeng Liu, Jinjian Yao, Koichi Matsuda, Yusuke Nakamura, Jinling Yu, Xiaorui Jiang, Shuzhen Sun, Qing Liu, Xiang Jiang, Qilong Song, Man Chen, Hong Yang, Feng Tang, Xiaowen Hu, Jing Wang, Ying Chang, Xingxing He, Yuan Chen, Jusheng Lin

**Affiliations:** 1 Institute of Liver Disease, Tongji Hospital, Tongji Medical College, Huazhong University of Science and Technology, Wuhan, Hubei Province, China; 2 The Third People's Hospital of Shenzhen, Shenzhen, Guangdong Province, China; 3 Department of Infection Disease, Union Hospital, Tongji Medical College, Huazhong University of Science and Technology, Wuhan, Hubei Province, China; 4 Department of Oncology, Tongji Hospital, Tongji Medical College, Huazhong University of Science and Technology, Wuhan, Hubei Province, China; 5 Qingdao Infectious Disease Hospital, Qingdao, Shandong Province, China; 6 Laboratory of Molecular Medicine, Human Genome Center, Institute of Medical Science, The University of Tokyo, Tokyo, Japan; South Texas Veterans Health Care System, United States of America

## Abstract

**Background:**

Human leukocyte antigen DP (*HLA-DP*) locus has been reported to be associated with hepatitis B virus (HBV) infection in populations of Japan and Thailand. We aimed to examine whether the association can be replicated in Han Chinese populations.

**Methodology/Principal Findings:**

Two *HLA-DP* variants rs2395309 and rs9277535 (the most strongly associated SNPs from each *HLA-DP* locus) were genotyped in three independent Han cohorts consisting of 2 805 cases and 1 796 controls. By using logistic regression analysis, these two SNPs in the *HLA-DPA1* and *HLA-DPB1* genes were significantly associated with HBV infection in Han Chinese populations (*P* = 0.021∼3.36×10^−8^ at rs2395309; *P* = 8.37×10^−3^∼2.68×10^−10^ at rs9277535). In addition, the genotype distributions of both sites (rs2395309 and rs9277535) were clearly different between southern and northern Chinese population (*P* = 8.95×10^−5^ at rs2395309; *P* = 1.64×10^−9^ at rs9277535). By using asymptomatic HBV carrier as control group, our study showed that there were no associations of two *HLA-DP* variants with HBV progression (*P* = 0.305∼0.822 and 0.163∼0.881 in southern Chinese population, respectively; *P* = 0.097∼0.697 and 0.198∼0.615 in northern Chinese population, respectively).

**Conclusions:**

Our results confirmed that two SNPs (rs2395309 and rs9277535) in the *HLA-DP* loci were strongly associated with HBV infection in southern and northern Han Chinese populations, but not with HBV progression.

## Introduction

More than 2 billion people have been infected with the hepatitis B virus (HBV) worldwide, of which 350 million are chronic carriers and about 600 000 die annually of HBV-related acute or chronic liver disease [1]. Although many individuals eventually achieve a state of nonreplicative infection, the prolonged immunologic response to infection leads to the development of cirrhosis, liver failure, or hepatocellular carcinoma (HCC) in up to 40% of patients [2]. In China, where HBV infection is endemic, there are estimated 93 million HBV carriers, and among them 30 million are patients with chronic hepatitis B [3]. Multiple causes influence the risk of chronic HBV infection in china, for example, age, gender, viral genotype, ethnicity, variation in genes of the immune system and so on [4].

Several polymorphisms of the *HLA* loci have been reported for hepatitis B virus infection [5], [6]. A study in Gambian found that the allele DRB1*1302 was associated with the clearance of the virus [7]. Hepatitis B virus persistence and disease chronicity were associated with *HLA-DQA*1*0501 and *HLA-DQB*1*0301 in Chinese [8] and with *HLA-DR*9 in Koreans [9]. Although the association between common diseases and these *HLA* (or non-*HLA*) genes has become increasingly evident [10], their results are conflicting among the studies, and have not been confirmed by other investigators [11].

A recent study found that the *HLA-DP* locus was associated with chronic hepatitis B in Japanese and Thais [12]. As the frequencies of these *HLA-DP* alleles in Chinese populations were similar to those in Japanese populations, it would be necessary to confirm whether there was the association between the *HLA-DP* genetic variation and HBV infection in Chinese populations. To this end, we selected the most strongly associated SNPs (the previous GWAS results) from each *HLA-DP* locus (rs9277535 at the *HLA-DPB1* and rs2395309 at the *HLA-DPA1*, respectively) and genotyped these two polymorphisms in a population-based case-control study of Chinese Hans, including 2 805 cases and 1 796 controls from Hubei province (Central China), Shandong province (North China) and Guangdong province (South China).

## Materials and Methods

### Ethic statement

The study was approved by the local research ethics committee (REC) at the Tongji Hospital of Huazhong University of Science and Technology in accordance with the principle of the Helsinki Declaration II. All written informed consent documents from each participant were obtained during the enrollment phase.

### Study subjects

A total of 4 601 unrelated Han Chinese were recruited in this study between September 2007 and June 2011. All subjects were divided into six groups: a) HBV clearance group(Clear); b) Healthy control group(Health); c) Persistent asymptomatic HBV carriers group(AsC); d) Chronic active hepatitis B group(CHB); e) HBV-related liver cirrhosis group(LC); and f) HBV-related heptocellular carcinoma group(HCC). The diagnostic criteria for study inclusion were listed in Table S1, which had been described in the previous publication [13], [14]. All individuals were gathered from three Han Chinese cohorts. First, we recruited 2 280 subjects from Tongji hostital and Union hospital in Wuhan, Hubei province. Second, we gathered additional 1 304 subjects from The Affiliated Hospital of Binzhou Medical College and Qingdao Infectious Disease Hospital in Shandong province, 1 017 subjects from Shenzhen Third People's Hospital, Shenzhen Fourth People's Hospital and Shenzhen Sixth People's Hospital in Guangdong province.

A uniform questionnaire was used at three enrollment sites and recorded self-report of risk factors for HBV transmission, family history of HBV infection, past and current smoking, alcohol ingestion, etc. The demographic information included gender, birth-date, birthplace, and past and current residency.

### DNA Isolation and Genotyping

Genomic DNA was isolated from peripheral whole blood using TIANamp blood DNA kit (Tiangen Biotech [Beijing] Co., Ltd., China). The concentration and purity of the DNA were determined with a NanoDrop spectrophotometer and diluted to a final concentration of 8 ng/µL. The genotyping of genetic polymorphisms was performed via the TaqMan method according to the protocol of TaqMan® SNP Genotyping Assays (Applied Biosystems, California, USA). Allelic category was measured automatically using the Sequence Detection System 2.3 software (Applied Biosystems) according to the intensity of VIC and FAM dye. To detect these SNPs (rs2395309 and rs9277535), we customized the TaqMan® MGB Probe as well as the primers for PCR amplification (Table S2.).

### Statistical analysis

Statistical analysis was conducted by using haploview 4.2, Arlequin 3.5, Stata10.0 and SPSS 17.0 softwares. Linkage disequilibrium was assessed by the haploview 4.2 softwares using frequencies obtained from the Health group. The (Bayesian) ELB algorithm was used to infer haplotypes by using Arlequin 3.5. The Hard-Weinberg equilibrium of alleles and population pairwise comparisons were also evaluated by using Arlequin 3.5 [15]. A meta-analysis of all studies was performed for each SNP associated with chronic hepatitis B by using Stata10.0 softwares. Odds ratios (ORs) and 95% confidence intervals (CIs) were calculated on the basis of the binary logistic regression analysis (adjustment for gender and age). The strength of association between the genotypes or alleles and HBV infection was estimated by using SPSS 17.0 softwares. A best-fit model was constructed by means of comparisons with other models. Values of P<0.05 were considered statistically significant.

## Results

### Hardy-Weinberg equilibrium test

Hardy-Weinberg equilibrium was estimated by Fisher's exact test using Arlequin 3.5 software. There was no significant difference between observed and expected frequencies of each genotype in these involved populations (P>0.05). This result indicated that these populations had a relatively stable genetic background and were suitable for further genetic statistical analysis.

### The clinic and demographic characteristics

The clinical and demographic characteristics of the case-control study were summarized in Table 1, including gender, age, drinkers, serum total bilirubin level (T-Bil), HBV-DNA load, alanine transaminase (ALT) and serum markers of hepatitis B virus. There was no significant difference in the percentage of hepatitis B e antigen (HBeAg) positive (*P* = 0.10) between asymptomatic HBV carriers (17.1%) and the patients of chronic hepatitis B group (20.2%). In addition, there was more alcohol consumption in patients (*P*<0.05) with HBV-related liver cirrhosis group (24.3%) and HBV-related heptocellular carcinoma group (30.5%) than those in HBV clearance group (10.2%) and healthy control group (8.9%). The difference in the alcohol consumption status was due to few drinkers in Chinese female population. Although an effort was made to obtain a good match on age and sex, there were more men in four case groups (averaged 73.5%) than those in HBV clearance group (51.6%, *P*<0.05) and healthy control group (47.5%, *P*<0.05).

**Table 1:** Clinical characteristics of study subjects.

Characteristics	Health(n = 962)	Clear(n = 834)	AsC(n = 910)	CHB(n = 964)	LC(n = 544)	HCC(n = 387)
Gender, no. (%)						
Male	454(47.5)	421(51.6)	519(58.4)	765(81.0)	435(80.3)	342(88.8)
Female	501(52.5)	395(48.4)	370(41.6)	180(19.0)	107(19.7)	43(11.2)
Age (years), mean(sd)	51.70±10.96	51.48±11.04	45.64±10.93	38.45±10.73	48.30±11.02	49.82±10.01
Drinkers, no. (%)	86(8.9)	85(10.2)	102(11.2)	179(18.6)	132(24.3)	118(30.5)
HBsAg	All−	All−	All+	All+	All+	All+
Anti-HBs IgG	All−	All+	All−	All−	All−	All−
HBeAg-positive,no. (%)	All−	All−	156(17.1)	195(20.2)	64(11.8)	39(10.1)
Anti-HBc IgG, no. (%)	All−	All+	All+	All+	All+	All+
Family history, no. (%)	No	No	36(4.0)	142(14.7)	69(12.7)	77(19.9)
ALT(U/L)	No	No	19.74±15.09	447.53±402.71	101.57±119.90	83.64±86.17
TBil (µmol/L)	No	No	11.71±9.01	161.55±171.06	124.19±125.86	69.24±65.74
HBV-DNA (copy/ml)	No	No	No	3.23E7±4.76E7	1.38E6±5.16E6	3.72E6±1.13E7

‘No’ means non-detected.

‘Drinkers’ was defined as alcohol consumption of >40 g/week, which included occasional drinkers and daily drinkers.

Abbreviations: Clear, HBV clearance group; Health, Healthy control group; AsC, Asymptomatic HBV carriers group; CHB, Chronic active hepatitis B group; LC,HBV-related liver cirrhosis group; HCC, HBV-related heptocellular carcinoma group;

### Population pairwise comparisons and grouping of subjects

To explore whether differences in susceptibility loci were caused by the disease or by genetic background between populations, we first needed to determine which populations should be compared with each other, and whether there were populations that could be lumped together to simplify statistical analysis. To this end, we performed population pairwise comparisons **F_ST_** testing between each population using Arlequin 3.5 software. The principle of population pairwise comparisons states that: if there is no difference in heredity between two populations, the data permuting of genotypes or haplotypes between two populations should not cause a significant difference, which can be evaluated by **F_ST_** *P* value (*P*>0.05). According to the results shown in Table 2, we could infer that Shandong population had greater difference than Hubei population or Guangdong population in genetic background (*P*<0.0001). As the pairwise comparisons for Hubei population and Guangdong population were not significantly different (*P* = 0.191), and both of them were the same geographic position (southern of china) [16], we had determined to merge Hubei population and Guangdong population into southern Chinese population. Meanwhile, Shandong population was taken as northern Chinese population. Furthermore, in order to identify whether the two polymorphisms were associated with HBV infection or clearance, we combined all the types of HBV infection populations into one group by using the healthy group or clearance group as the reference.

**Table 2:** Matrix of significant F_ST_ *P* values among populations.

Populations	Hubei	Guangdong	Shandong
Hubei	*		
Guangdong	0.191	*	
Shandong	**<0.0001**	**<0.0001**	*

Population pairwise comparisons **F_ST_** tests were performed between pairs of groups using Arlequin 3.5software. Statistically significant values are in shown in bold.

Abbreviations: Hubei, Hubei populations; Guangdong, Guangdong populations; Shandong, Shandong populations.

### Logistic regression analysis of the *HLA-DP* loci polymorphisms

Then, to investigate which genotypic models were significantly associated with the various outcomes, we conducted comparisons of four models (Multiplicative model, Additive model, Dominant model and Recessive model) in southern and northern Chinese populations respectively (data not show). For the four models, the best-fit genotypic effect of these two SNPs (rs2395309 and rs9277535) was observed in the dominant model which was the protective genotype AA and AG (see Table 3). After compared with the Healthy control group, both single nucleotide polymorphism (SNP) sites (rs2395309 and rs9277535) showed associations with HBV infection in southern Chinese population (Odds ratio [OR] = 0.57; 95% Confidence intervals [CI] :0.47, 0.70; *P* = 3.36×10^−8^ at rs2395309; OR = 0.52; 95% CI :0.43, 0.64; *P* = 2.68×10^−10^ at rs9277535), as well as in northern Chinese population (OR = 0.50; 95% CI :0.35, 0.71; *P* = 1.23×10^−4^ at rs2395309; OR = 0.50; 95% CI :0.36, 0.68; *P* = 1.74×10^−5^ at rs9277535). And, interestingly, *HLA-DP* rs2395309 and rs9277535 sites also showed a strong protective effect for HBV clearance not only in southern Chinese population (OR = 1.31; 95% CI :1.17, 1.45; *P* = 9.63×10^−7^ at rs2395309; OR = 1.33; 95% CI :1.20, 1.49; *P* = 1.67×10^−7^ at rs9277535) but also in northern Chinese population (OR = 1.20; 95% CI :1.03, 1.40; *P* = 0.021 at rs2395309; OR = 1.26; 95% CI :1.06, 1.49; *P* = 8.37×10^−3^ at rs9277535). As shown in Table 3, notably, the genotype distributions of both sites (rs2395309 and rs9277535) were clearly different between southern and northern Healthy populations (*P* values = 8.95×10^−5^ and 1.64×10^−9^, respectively. *P* values of Pearson's x^2^ test for allele model). The two minor-allele frequencies (MAF) in both Healthy populations (southern and northern Han Chinese) were 30.1% vs 38.8% at rs2395309, 38.1% vs 52.2% at rs9277535. In addition, to decrease the bias of sex and age in population sampling, we further conducted the stratified analysis for sex and age. As presented in Table S3, male and female patients showed different associations with HBV diseases in these two SNPs (rs2395309 and rs9277535). Specially, in the northern Chinese population, this difference was notable between male patients and female patients. Furthermore, in the stratified analysis of age, most cases were no significant differences in genotype distributions of two SNPs sites between patients with age≤45 years and patients with age>45 years (Table S4.).

**Table 3:** Associations of two SNPs (rs2395309, rs9277535) with HBV infection and clearance in Han Chinese populations.

	South of china		North of china	
	Control group	Case group	Control group	Case group
*HLA-DPB1* (rs2395309)- dominant model (AA+AGvsGG)				
AA/AG/GG	57/234/288†	112/709/1367‡	52/193/138†	63/249/302‡
*P* value OR (95%CI)	Reference	3.36×10^−8^ 0.57 (0.47,0.70)	Reference	1.23×10^−4^ 0.50 (0.35,0.71)
AA/AG/GG	112/709/1367‡	35/235/257∥	63/249/302‡	56/130/121∥
*P* value OR (95%CI)	Reference	9.63×10^−7^ 1.31 (1.17,1.45)	Reference	0.021 1.20 (1.03,1.40)
*HLA-DPA1* (rs9277535)- dominant model (AA+AGvsGG)				
AA/AG/GG	80/277/216†	177/830/1195‡	97/203/80†	118/287/206‡
*P* value OR (95%CI)	Reference	2.68×10^−10^ 0.52 (0.43,0.64)	Reference	1.74×10^−5^ 0.50 (0.36,0.68)
AA/AG/GG	177/830/1195‡	67/251/208∥	118/287/206‡	67/165/75∥
*P* value OR (95%CI)	Reference	1.67×10^−7^ 1.33 (1.20,1.49)	Reference	8.37×10^−3^ 1.26 (1.06,1.49

† Healthy control group.

∥ HBV clearance group.

‡ HBV infection groups, including Asymptomatic HBV carriers, Chronic active hepatitis B group, HBV-related liver cirrhosis group, HBV-related heptocellular carcinoma group.

The *P* values, odds ratios (OR), and 95% confidence intervals (CI) were calculated on the basis of the binary logistic regression analysis, adjusted for sex and age.

### Associations of the *HLA-DP* loci polymorphisms with HBV progression

Considering the function of *HLA-DP* molecules, we were interested in the possible association between the polymorphisms in *HLA-DP* gene and the disease progression of chronic hepatitis B. To test our prediction, we further analysed the difference in two SNPs genotype distributions by using asymptomatic HBV carrier as control group. Unfortunately, there were not associations in chronic active hepatitis B group (OR = 1.03; 95% CI : 0.79, 1.34; *P* = 0.822 at rs2395309; OR = 0.92; 95% CI : 0.71, 1.18; *P* = 0.501 at rs9277535, in southern Chinese population; OR = 0.92; 95% CI : 0.62, 1.38; *P* = 0.697 at rs2395309; OR = 1.33; 95% CI : 0.86, 2.06; *P* = 0.198 at rs9277535, in northern Chinese population), HBV-related liver cirrhosis group (OR = 1.11; 95% CI : 0.82, 1.52; *P* = 0.499 at rs2395309; OR = 1.24; 95% CI : 0.92, 1.67; *P* = 0.163 at rs9277535, in southern Chinese population; OR = 0.74; 95% CI : 0.48, 1.16; *P* = 0.189 at rs2395309; OR = 1.29; 95% CI : 0.81, 2.06; *P* = 0.286 at rs9277535, in northern Chinese population) and HBV-related heptocellular carcinoma group(OR = 0.85; 95% CI : 0.63, 1.16; *P* = 0.305 at rs2395309; OR = 0.98; 95% CI : 0.73, 1.31; *P* = 0.881 at rs9277535, in southern Chinese population; OR = 0.56; 95% CI : 0.28, 1.11; *P* = 0.097 at rs2395309; OR = 0.84; 95% CI : 0.42, 1.68; *P* = 0.615 at rs9277535, in northern Chinese population), compared with asymptomatic HBV group(Table S5.).

### Associations of the *HLA-DP* loci polymorphisms with clinical factors

In order to analyze the associations between two SNPs and clinical factors (HBV-DNA load, ALT and TB), we used the independent-sample Kolmogorov-Smirnov t test in CHB group, LC group and HCC group. Although the GG patients have a higher mean on the HBV-DNA load, no significant difference was found between patients of different genotypes (see Fig. S1). In the analysis of ALT, the associations between two SNPs and the ALT level only be found in HBV-related liver cirrhosis group (*P* = 0.002 at rs2395309; *P* = 0.009 at rs9277535), rather than in other groups. Meanwhile, for the associations of the TB level, there was no difference between GG patients and AG+AA patients (*P*>0.05 in each group).

### Results of the Haplotype analysis and Meta-analysis

**Figure 1:**
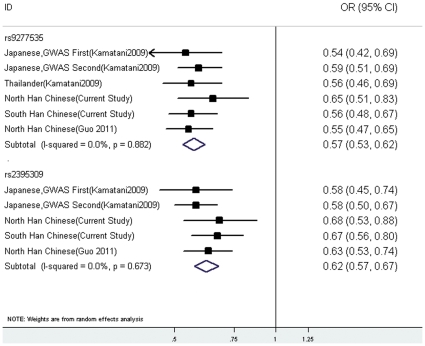
Meta-analasis of the rs9277535 and rs2395309. The meta-analysis combined with the results of previous studies, including more than 2,243 cases and 4,137 controls. Each effect size is shown with its confidence interval. Abbreviations: p, *P* heterogeneity value; OR, odds ratios; 95%CI, 95% confidence interval.

To further understand the contributions of these loci to HBV susceptibility, two-locus haplotypes were constructed for two SNPs rs2395309 and rs9277535 (Table 4.). Pairwise linkage disequilibrium (LD) analyses performed using all individuals from the health group showed that rs2395309 and rs9277535 SNPs were in LD with each other (D′ = 0.57, r^2^ = 0.23 in southern Chinese population; D′ = 0.58, r^2^ = 0.20 in northern Chinese population). In trying to derive HBV infection-specific haplotypes, the haplotype frequencies of two SNPs (rs2395309 and rs9277535) were evaluated in both Chinese populations. Four haplotypes were observed, and among them three haplotypes had frequencies more than 5% (Table 4.). Compared with protective A-A haplotype homozygotes, only G-G haplotype homozygotes had a significant increased risk for HBV infection (*P* value and odds ratios were shown in Table 4). Then, we summarized a meta-analysis combined with the results of related studies [12], [17], including more than 2,243 cases and 4,137 controls. As shown in Figure 1 and Table S6, these odds ratios were quite similar among the three ethnic groups (Japanese, Thais and Chinese) and no heterogeneity was observed (*P* het = 0.673 at rs2395309; *P* het = 0.882 at rs 9577535).

**Table 4:** Results of the association test for two SNPs(rs2395309,rs9277535) haplotypes in Han Chinese populations.

South of china						
Haplotype	Health(2n = 1106)	Clear(2n = 1048)	AsC(2n = 1342)	CHB(2n = 1486)	LC(2n = 754)	HCC(2n = 632)
A-A	247(22.3)	205(17.0)	227(16.8)	238(16.0)	122(16.2)	92(14.5)
A-G	88(8.0)	97(11.8)	62(4.6)	95(6.4)	36(4.8)	23(3.7)
G-A	173(15.6)	177(19.5)	140(10.4)	143(9.6)	95(12.6)	78(12.4)
G-G	598(54.1)	569(51.7)	913(68.1)	1010(68.0)	501(66.4)	439(69.4)
*P* value⊩	Reference		1.47×10^−6^	6.47×10^−8^	2.53×10^−5^	6.07×10^−7^
OR (95%CI)			0.60 (0.49,0.74)	0.57 (0.47,0.70)	0.59 (0.46,0.76)	0.51 (0.39,0.66)
*P* value╢		Reference	7.35×10^−4^	8.92×10^−5^	2.45×10^−3^	1.06×10^−4^
OR (95%CI)			1.45 (1.17,1.80)	1.53 (1.24,1.89)	1.48 (1.15,1.91)	1.72 (1.31,2.27)

North of china						
Haplotype	Health(2n = 734)	Clear(2n = 608)	AsC (2n = 422)	CHB (2n = 378)	LC(2n = 300)	HCC(2n = 100)
A-A	226(30.8)	200(31.5)	118(28.0)	103(27.3)	71(23.6)	21(20.5)
A-G	55(7.5)	41(8.1)	27(6.4)	18(4.8)	16(5.4)	4(3.5)
G-A	157(21.4)	98(17.5)	66(15.6)	60(15.9)	62(20.7)	15(15.4)
G-G	296(40.3)	269(42.9)	211(50.0)	197(52.1)	151(50.3)	60(60.5)
*P* value⊩	Reference		0.032	0.012	0.004	0.003
OR (95%CI)			0.73 (0.55,0.97)	0.68 (0.51,0.92)	0.62 (0.44,0.86)	0.46 (0.27,0.78)
*P* value╢		Reference	0.054	0.021	0.007	0.005
OR (95%CI)			1.33 (0.99,1.78)	1.42 (1.05,1.92)	1.58 (1.13,2.21)	2.12 (1.25,3.61)

⊩ Two SNPs haplotypes G-G, A-A in Health group compared with those in HBV infection groups.

╢ Two SNPs haplotypes G-G, A-A in HBV infection groups compared with those in Clearance group.

The *P* values, odds ratios (OR), and 95% confidence intervals (CI) were calculated by Pearson Chi-Square test.

Abbreviations: Clear, HBV clearance group; Health, Healthy control group; AsC, Asymptomatic HBV carriers group; CHB, Chronic active hepatitis B group; LC,HBV-related liver cirrhosis group; HCC, HBV-related heptocellular carcinoma group; OR, odds ratio; CI, confidence interval.

## Discussion

In this analysis, we confirmed that two SNPs sites (rs2395309 and rs9277535) in the *HLA-DPA1* and *HLA-DPB1* genes were significantly associated with HBV infection in southern and northern Han Chinese populations. Again, our haplotype analysis showed the frequency of G-G haplotype had a significant increase in the HBV infected populations, as compared with the healthy control group or HBV clearance group. As a result, we inferred that these persons with G-G haplotype have a higher risk of HBV infection than those persons with A-A haplotype. Meanwhile, the A-A haplotype could be strongly predictive for HBV clearance in HBV infection populations. Although our manuscript suggested that the genotype distributions of both sites (rs2395309 and rs9277535) were different between southern and northern Chinese population, the frequencies of two protective alleles A in Chinese populations were also similar to those in Asian populations, compared with European and Central American populations (data from public databases, HapMap). The results of the genetic association in our study were consistent with the previous study [12]. Hence, we could confirm that the polymorphisms of *HLA-DPA1* and *HLA-DPB1* gene play a very important role in chronic hepatitis B virus infection in southern and northern Han Chinese populations.

It has been well documented that men are more likely than women to be infected with HBV and develop liver cirrhosis and hepatocellular carcinoma [18], [19]. The reasons for the gender distinction between HBV populations and health populations are complex, including occupation, alcohol drinking, tobacco smoking, family history of HBV infection and so on. Some previous reports suggested that sex hormones might interact with HBV in the infection process and lead to a dominant sex disparity in HBV populations. Naugler et al. [20] found that estrogen-mediated inhibition of interleukin-6 production by Kupffer cells reduced the risk of liver cancer in females. Wang et al. [21] study demonstrated that the androgen pathway could increase the transcription of HBV through direct binding to the androgen-responsive element sites in viral enhancer. Consequently, to decrease the bias of sex in population sampling, we further conducted the stratified analysis for sex. Although we found that male and female northern Chinese showed a different susceptibility to HBV infection, it only had 25% and 21% statistical power to detect these ORs of 0.73 and 0.74, which may lead to the false-negative results of rs2395309 and rs9277535 in northern female Chinese. The small sample for female HBV patients in this study might be the major reason for the non-significant associations in female Chinese. Hence, we only concluded that the genetic variants of *HLA-DPA1* and *HLA-DPB1* loci differ slightly between male and female Chinese, and the reasons why there is different between male and female for HBV infection need to be further studied.

And indeed, by consulting previous studies [22], [23], we found that there are different distributions in some *HLA* alleles among Han Chinese populations. For instance, HLA-DRB1*0301 [8], a risk-allele with respect to chronic HBV infection in Han Chinese, markedly has higher frequency in southern Han Chinese population than those in northern Han Chinese population. Since the frequency distribution of *HLA-DP* alleles were barely reported in China, it could be inferred only indirectly that there were also different distributions at *HLA-DP* alleles between two Han Chinese populations. And, it was the difference that led to the distinct distributions of both SNPs (rs9277535 and rs2395309) between southern and northern Han Chinese population. Nevertheless, this explanations why the distributions of the *HLA* alleles (or SNPs) differed between Han Chinese populations were complicated, such as evolution and migration history of the Chinese population [24], [25], [26], MHC-based mate choice [27], pathogen-driven selection at *HLA* alleles [28], [29] and so on. Taking into account the different distributions of HBV genotypes [30] and HBV carrier rate [31] in China, as well as recent studies [12], [17] and our results, we deduced that the mechanism of pathogen-driven selection (HBV and/or other pathogens) might be the leading cause of the different distributions at *HLA-DP* alleles between two Han Chinese populations.

Moreover, after infection with hepatitis B virus (HBV), the host's inflammatory immune response induces hepatocellular damage and is followed by the pathogenesis of liver cirrhosis and cancer [32]. Liver cancer arises most frequently in the setting of chronic liver inflammation [33]. Considering the function of *HLA-DP* molecules, HBV antigen presentation on *HLA-DP* molecules may be critical for virus elimination and has an important role in the progression of hepatitis B [34]. Therefore, we further analysed the possible association between the polymorphisms in *HLA-DP* gene and the disease progression of chronic hepatitis B. Unfortunately, compared with asymptomatic HBV carrier, there were no associations in chronic active hepatitis B group, HBV-related liver cirrhosis group and HBV-related hepatocellular carcinoma group. Although chronic HBV infection is the most important cause of HCC worldwide and contributes to at least 70% of cases of HCC in Asian-Africa [35], only a tiny fraction of chronic HBV carriers develop HCC in their lifetime [36]. It is suggested that the risk of HCC is caused by a complex interplay between multiple genetic and environmental factors. Recently, Zhang et al. have conducted the first liver GWAS for HCC in Chinese ancestry and identified a single susceptibility locus in the UBE4B-KIF1B-PGD region on 1p36.22 [37]. Since the region involve in these aspects of vesicles transport, cell apoptosis, DNA repair, and other intracellular pathways, it seems likely that different genes play disparate roles in HBV infection and HBV progression. For example, immune pathway (*HLA-DP* or other genes) is the primary cause of HBV infection, but intracellular pathway (Ubiquitin or other pathways) is the major reason of HBV progression. Thus, by combining our results with the aforementioned discussion, we inferred that the polymorphisms in *HLA-DPA1* and *HLA-DPB1* gene influence the infection of HBV in Chinese populations, rather than the progression of HBV disease.

Since the early 1970s [38], classical human leukocyte antigen loci have stood out as the leading candidates for infectious disease susceptibility. The classical *HLA* loci are the class I (*HLA-A*, *-B*, *-C*, *-E*, *-F*, and -*G*) and class II (*HLA-DR*, *-DQ*, *-DM*, and *-DP*) molecules. *HLA* class II molecules are the central part in the immune system by presenting peptides to the antigen receptor of CD4+ T cells [39]. Antigen presentation is not only crucial for the regulation of protective immune responses against invading pathogens, but also necessary for the maintenance of self-tolerance. It is therefore perhaps not surprising to find that the human MHC class II gene region holds the largest number, and some of the longest recognised, associations with a autoimmune, inflammatory and infectious diseases [40], [41]. Although *HLA-DPs* have a structure similar to other classical *HLA* class II molecules, *HLA-DP* molecule roles in the immune response have not been well characterized until now. In a previous study, Hirayama et al. [42] indicated that the *HLA* class II genes for the *HLA-DR-DQ* alleles were associated with protection against early changes in liver fibrosis, whereas *HLA-DP* alleles were associated with protection from the late phase of schistosomal hepatic fibrosis. Owing to lack of replication of the previously report, more studies are essential to provide conclusive genetic and functional evidence to support a role for *HLA-DP* in HBV disease susceptibility.

In summary, in this multicenter case-control study, we have confirmed that the G alleles of two SNPs sties in the *HLA-DPA1* and *HLA-DPB1* were significantly associated with hepatitis B virus (HBV) infection in Han Chinese populations, and both A alleles (rs2395309 and rs9277535) also showed a strong protective effect for HBV clearance. Furthermore, we found that the genotype distributions of both sites (rs2395309 and rs9277535) were clearly different between southern and northern Han Chinese population. By using asymptomatic HBV carrier as control group, our study showed that there were no associations of *HLA-DP* variants (rs2395309 and rs9277535) with HBV progression. Although HBV disease is not determined solely by genetic factors, the experimental results offer the foundation for further study of genetic variations in the *HLA-DPA1* and *HLA-DPB1* for the prevention and therapy of chronic HBV infection.

## Supporting Information

Figure S1 **Associations of these two SNPs (rs2395309, rs9277535) genotypes with HBV DNA levels.** *P* values of independent-sample Kolmogorov-Smirnov t test for dominant model (AA+AG vs GG). Abbreviations:SNPs, single nucleotide polymorphisms.
(TIF)

Table S1 **Diagnosis criteria for Healthy control group (Health), HBV clearance group (Clear), Asymptomatic chronic HBV carriers group (AsC), Chronic active hepatitis B group (CHB), HBV-related liver cirrhosis group (LC) and HBV-related heptocellular carcinoma group (HCC).**
(DOC)

Table S2 **TaqMan probes and Primers for two SNPs (rs2395309 and rs9277535).**
(DOC)

Table S3 **The stratified analysis of gender between two SNPs (rs2395309, rs9277535) genotypes and different populations.** Male and female patients showed different genotype distributions in these two SNPs (rs2395309 and rs9277535), specially in the northern Chinese population. The *P* values, odds ratios (OR), and 95% confidence intervals (CI) were calculated on the basis of the binary logistic regression analysis, adjusted for age.
(DOC)

Table S4 **The stratified analysis of age between two SNPs (rs2395309, rs9277535) genotypes in south Chinese population and north Chinese population.** Most cases were no significant difference in genotype distributions of two SNPs sites between patients with age≤45 years and patients with age>45 years. The *P* values, odds ratios (OR), and 95% confidence intervals (CI) were calculated on the basis of the binary logistic regression analysis, adjusted for sex.
(DOC)

Table S5 **Associations of two SNPs (rs2395309, rs9277535) with HBV progression in Han Chinese populations.** Compared with asymptomatic HBV group, those two sites (rs2395309 and rs9277535) in HLA-DPA1 or HLA-DPB1 gene had no associations with the chronic active hepatitis B, the HBV-related liver cirrhosis, and the HBV-related heptocellular carcinoma in southern and northern Chinese population.
(DOC)

Table S6 **A Meta-analysis for previous study and current study ( more than 2,243 cases and 4,137 controls).** Genotype distributions of rs9277535 and rs2395309 in three ethnic groups (Japanese, Thais, Chinese) between healthy control group and chronic active hepatitis B group. *P* values of Pearson's x^2^ test for allele model. Odds ratios (OR) and 95% confidence intervals (CI) of minor allele from two-by-two allele frequency table.
(DOC)
