# Supplementary figures and images for: Associations of HLA-DP Variants with Hepatitis B Virus Infection in Southern and Northern Han Chinese Populations: A Multicenter Case-Control Study
Source: PLoS One. 2011 Aug 31;6(8):e24221. doi: 10.1371/journal.pone.0024221 (PMC3164164; doi:10.1371/journal.pone.0024221)

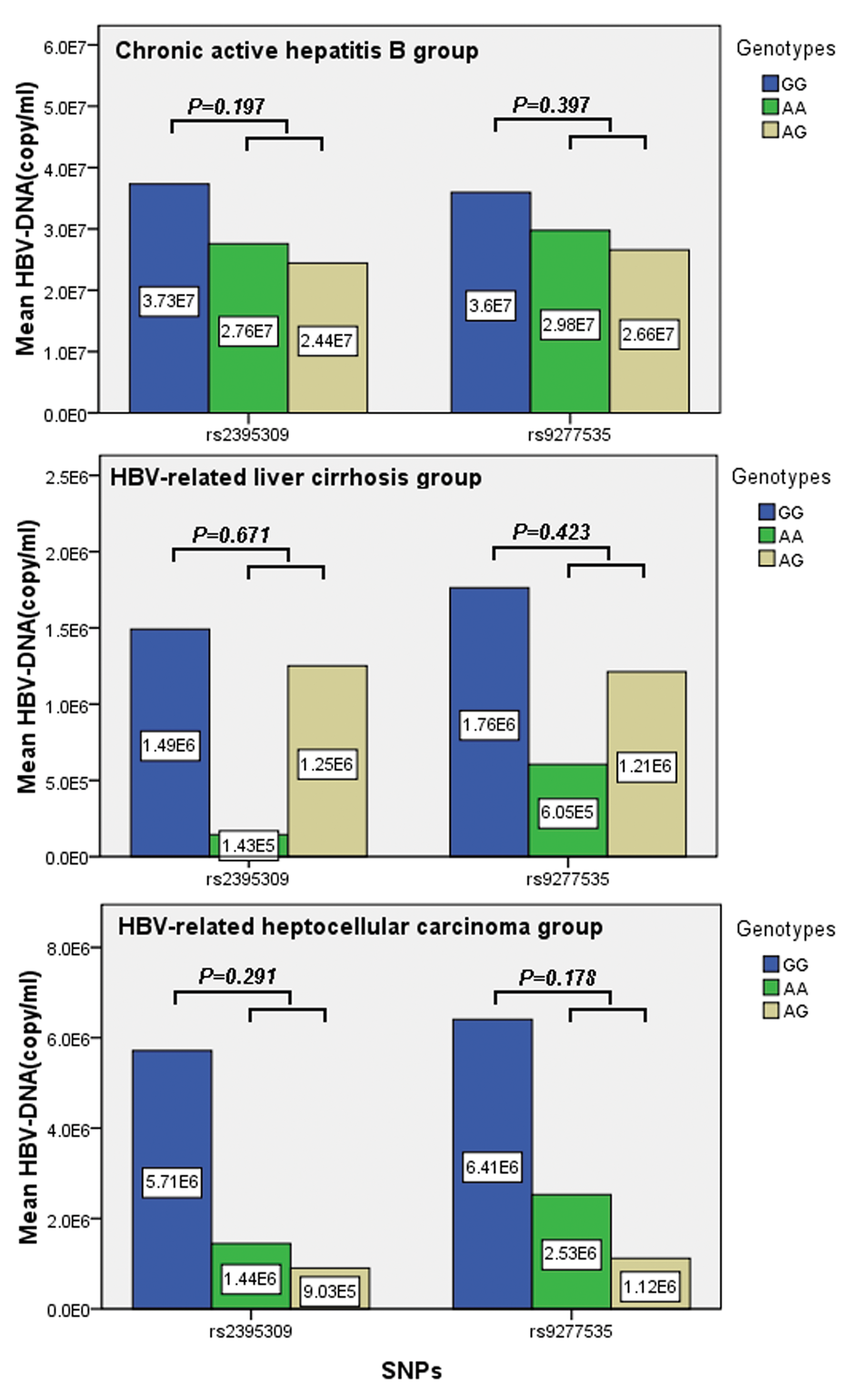

Supplement: Figure S1 — Associations of these two SNPs (rs2395309, rs9277535) genotypes with HBV DNA levels. P values of independent-sample Kolmogorov-Smirnov t test for dominant model (AA+AG vs GG). Abbreviations:SNPs, single nucleotide polymorphisms. (TIF) [file pone.0024221.s001.tif]
