# Supplementary material for: Associations of HLA-DP Variants with Hepatitis B Virus Infection in Southern and Northern Han Chinese Populations: A Multicenter Case-Control Study
Source: PLoS One. 2011 Aug 31;6(8):e24221. doi: 10.1371/journal.pone.0024221 (PMC3164164; doi:10.1371/journal.pone.0024221)
Supplement: Table S1 — Diagnosis criteria for Healthy control group (Health), HBV clearance group (Clear), Asymptomatic chronic HBV carriers group (AsC), Chronic active hepatitis B group (CHB), HBV-related liver cirrhosis group (LC) and HBV-related heptocellular carcinoma group (HCC). (DOC) [file pone.0024221.s002.doc]

**Table S1.**

**Diagnosis criteria for Healthy control group(Health), HBV clearance group(Clear), Persistent asymptomatic HBV carriers group (AsC) ,** **Chronic active hepatitis B group (CHB),** **HBV-related liver cirrhosis group(LC) and** **HBV-related heptocellular carcinoma group(HCC).**

| Healthy control group(Health) |
| --- |
| 1. Anti-HBs, HBsAg and anti-HBc negative and no HBV vaccination history; |
| 2. Anti-HCV and HCV RNA negative; |
| 3. Anti-HDV and/or HDAg negative; |
| 4. ALT <40 and AST <45 IU/L; |
| 5. Age ≥ 35. |

| HBV clearance group(Clear) |
| --- |
| 1. HBsAg negative plus anti-HBs and anti-HBc positive; |
| 2. HBV-DNA negative, HDAg negative and/or anti-HDV negative; |
| 3. Anti HCV and HCV RNA negative; |
| 4. ALT <40 IU/L and AST <45 IU/L at enrollment; |
| 5. Age ≥ 35. |

| Persistent asymptomatic HBV carriers group(AsC) |
| --- |
| 1. Two positive tests for HBsAg and anti-HBcAg at least 6 months apart; |
| 2. Anti-HCV and HCV RNA negative; |
| 3. Anti-HDV and/or HDAg negative; |
| 4. ALT <40 and AST <45 IU/L for at least 12 or more months; |
| 5. No clinical symptoms of hepatitis, liver cirrhosis and liver cancer; |
| 6. Age ≥ 35. |

| Chronic active hepatitis B group(CHB) |
| --- |
| 1. Two positive tests for HBsAg and anti-HBcAg at least 6 months apart; |
| 2. Anti-HCV and HCV RNA negative; |
| 3. Anti-HDV and/or HDAg negative; |
| 4. ALT and/or AST levels greater than 2 times upper limits of normal range for testing hospital before or current(ALT and/orAST >80 IU/L); And, HBV-DNA Load>1000(copy/ml) |
| 5. No clinical evidence of liver cirrhosis. |

| HBV-related liver cirrhosis group(LC) |
| --- |
| 1. HBsAg and anti-HBc positive; |
| 2. Anti-HCV and HCV RNA negative; |
| 3. Anti-HDV and/or HDAg negative; |
| 4. LC confirmed by biopsy and sonography or CT or MRI; |
| 5. Liver cirrhosis with clinical presentation of gastroesophageal varication (3°) or a history of  bleeding, or ascites, or edema, or encephalopathy, or serum Albumin < 35 g/L, total bilirubin > 35  μmol/L. |

| HBV-related heptocellular carcinoma group(HCC) |
| --- |
| 1. HBsAg and anti-HBc positive; |
| 2. Anti-HCV and HCV RNA negative; |
| 3. Anti-HDV and/or HDAg negative; |
| 4. HCC confirmed by biopsy or elevated AFP and sonography or CT or MRI; |
