# Supplementary material for: Associations of HLA-DP Variants with Hepatitis B Virus Infection in Southern and Northern Han Chinese Populations: A Multicenter Case-Control Study
Source: PLoS One. 2011 Aug 31;6(8):e24221. doi: 10.1371/journal.pone.0024221 (PMC3164164; doi:10.1371/journal.pone.0024221)
Supplement: Table S2 — TaqMan probes and Primers for two SNPs (rs2395309 and rs9277535). (DOC) [file pone.0024221.s003.doc]

**Table S2. TaqMan probe and Primer for two SNPs（rs2395309, rs9277535）**.

|  | Rs2395309 | Rs9277535 |
| --- | --- | --- |
| Gene | *HLA-DPA1* | *HLA-DPB1* |
| Forward Primer | TGCATTATTTCCTGCCTCAGC | CTATTCTTAACTATTCAATGGTGAGCAGACT |
| Reverse Primer | GGTCTTATCCAGTTGGTGGTCAA | AATGATAAAACATGCTCTCAGTAAGGTATATG |
| MGB Probe 1 | FAM-TCTCACTTACAACTATCT –MGB | FAM-TAGGACCCGTATTCC-MGB |
| MGB Probe 2 | VIC-TCTCACTTATAACTATCTG-MGB | VIC-TAGGACCCATATTCC-MGB |
