# Supplementary material for: Associations of HLA-DP Variants with Hepatitis B Virus Infection in Southern and Northern Han Chinese Populations: A Multicenter Case-Control Study
Source: PLoS One. 2011 Aug 31;6(8):e24221. doi: 10.1371/journal.pone.0024221 (PMC3164164; doi:10.1371/journal.pone.0024221)
Supplement: Table S3 — The stratified analysis of gender between two SNPs (rs2395309, rs9277535) genotypes and different populations. Male and female patients showed different genotype distributions in these two SNPs (rs2395309 and rs9277535), specially in the northern Chinese population. The P values, odds ratios (OR), and 95% confidence intervals (CI) were calculated on the basis of the binary logistic regression analysis, adjusted for age. (DOC) [file pone.0024221.s004.doc]

**Table S3. The stratified analysis of gender between two SNPs（rs2395309, rs9277535）genotypes and different populations.**

|  | South of china | | North of china | |
| --- | --- | --- | --- | --- |
|  | Control group | Case group | Control group | Case group |
| *HLA-DPB1* (rs2395309)- dominant model (AA+AGvsGG) | | | | |
| Genotype(Male only) |  |  |  |  |
| AA/AG/GG | 33/105/123^†^ | 88/537/1018^‡^ | 27/97/71^†^ | 36/153/213^‡^ |
| *P* value OR (95%CI) | Reference | 2.48×10^-6^ 0.52（0.40,0.68） | Reference | 3.13×10^-4^ 0.43（0.28,0.68） |
| AA/AG/GG | 88/537/1018^‡^ | 15/115/124^║^ | 36/153/213^‡^ | 26/67/69^║^ |
| *P* value OR (95%CI) | Reference | 3.07×10^-4^ 1.30（1.13,1.50） | Reference | 0.044 1.23（1.01,1.51） |
| Genotype(Female only) |  |  |  |  |
| AA/AG/GG | 23/128/162^†^ | 21/150/300^‡^ | 25/96/67^†^ | 26/93/86^‡^ |
| *P* value OR (95%CI) | Reference | 0.003 0.63（0.47,0.85） | Reference | 0.305 0.73（0.40,1.34） |
| AA/AG/GG | 21/150/300^‡^ | 17/114/120^║^ | 26/93/86^‡^ | 29/62/51^║^ |
| *P* value OR (95%CI) | Reference | 0.001 1.32（1.12,1.55） | Reference | 0.254 1.14（0.91,1.45） |
| *HLA-DPA1* (rs9277535)- dominant model (AA+AGvsGG) | | | | |
| Genotype(Male only) |  |  |  |  |
| AA/AG/GG | 36/126/100^†^ | 131/626/893^‡^ | 48/107/40^†^ | 61/189/152^‡^ |
| *P* value OR (95%CI) | Reference | 2.38×10^-6^ 0.52（0.40,0.68） | Reference | 6.97×10^-6^ 0.37（0.24,0.57） |
| AA/AG/GG | 131/626/893^‡^ | 34/120/100^║^ | 61/189/152^‡^ | 33/89/40^║^ |
| *P* value OR (95%CI) | Reference | 3.21×10^-5^ 1.36（1.18,1.57） | Reference | 2.76×10^-3^ 1.41（1.13,1.76） |
| Genotype(Female only) |  |  |  |  |
| AA/AG/GG | 44/146/116^†^ | 42/173/264^‡^ | 49/96/40^†^ | 54/95/53^‡^ |
| *P* value OR (95%CI) | Reference | 2.04×10^-5^ 0.52（0.39,0.70） | Reference | 0.219 0.74（0.45,1.20） |
| AA/AG/GG | 42/173/264^‡^ | 29/122/99^║^ | 54/95/53^‡^ | 33/75/34^║^ |
| *P* value OR (95%CI) | Reference | 0.001 1.31（1.11,1.54） | Reference | 0.628 1.07（0.82,1.39） |

^†^ Healthy control group

^║^ HBV clearance group

^‡^ HBV infection groups, including Asymptomatic HBV carriers, Chronic active hepatitis B group, HBV-related liver cirrhosis group, HBV-related heptocellular carcinoma group
