# Supplementary material for: Associations of HLA-DP Variants with Hepatitis B Virus Infection in Southern and Northern Han Chinese Populations: A Multicenter Case-Control Study
Source: PLoS One. 2011 Aug 31;6(8):e24221. doi: 10.1371/journal.pone.0024221 (PMC3164164; doi:10.1371/journal.pone.0024221)
Supplement: Table S4 — The stratified analysis of age between two SNPs (rs2395309, rs9277535) genotypes in south Chinese population and north Chinese population. Most cases were no significant difference in genotype distributions of two SNPs sites between patients with age≤45 years and patients with age>45 years. The P values, odds ratios (OR), and 95% confidence intervals (CI) were calculated on the basis of the binary logistic regression analysis, adjusted for sex. (DOC) [file pone.0024221.s005.doc]

**Table S4. The stratified analysis of age between two SNPs（rs2395309, rs9277535）genotypes and different populations.**

|  | South of china | | North of china | |
| --- | --- | --- | --- | --- |
|  | Control group | Case group | Control group | Case group |
| *HLA-DPB1* (rs2395309)- dominant model (AA+AGvsGG) | | | | |
| Genotype(Age≤45 years) |  |  |  |  |
| AA/AG/GG | 20/96/125^†^ | 68/445/862^‡^ | 14/48/26^†^ | 30/109/136^‡^ |
| *P* value OR (95%CI) | Reference | 8.01×10^-4^ 1.30（1.12,1.52） | Reference | 0.02 0.41（0.19,0.90） |
| AA/AG/GG | 68/445/862^‡^ | 20/143/144^║^ | 30/109/136^‡^ | 8/29/21^║^ |
| *P* value OR (95%CI) | Reference | 9.18×10^-7^ 1.40（1.23,1.60） | Reference | 0.093 1.31（0.96,1.80） |
| Genotype(Age＞45 years) |  |  |  |  |
| AA/AG/GG | 37/138/163^†^ | 45/264/505^‡^ | 38/145/109^†^ | 33/140/166^‡^ |
| *P* value OR (95%CI) | Reference | 1.24×10^-5^ 1.35（1.18,1.54） | Reference | 1.81×10^-3^ 0.52（0.34,0.78） |
| AA/AG/GG | 45/264/505^‡^ | 15/92/113^║^ | 33/140/166^‡^ | 48/100/100^║^ |
| *P* value OR (95%CI) | Reference | 0.003 1.26（1.08,1.47） | Reference | 0.085 1.17（0.98,1.39） |
| *HLA-DPA1* (rs9277535)- dominant model (AA+AGvsGG) | | | | |
| Genotype(Age≤45 years) |  |  |  |  |
| AA/AG/GG | 29/120/88^†^ | 115/508/758^‡^ | 25/53/15^†^ | 55/134/84^‡^ |
| *P* value OR (95%CI) | Reference | 9.31×10^-5^ 0.54（0.39,0.73） | Reference | 8.45×10^-3^ 0.42（0.22,0.80） |
| AA/AG/GG | 115/508/758^‡^ | 40/146/119^║^ | 55/134/84^‡^ | 15/30/15^║^ |
| *P* value OR (95%CI) | Reference | 5.37×10^-6^ 1.37（1.20,1.57） | Reference | 0.384 1.17（0.82,1.65） |
| Genotype(Age＞45 years) |  |  |  |  |
| AA/AG/GG | 51/157/128^†^ | 62/322/437^‡^ | 72/150/65^†^ | 63/153/122^‡^ |
| *P* value OR (95%CI) | Reference | 2.72×10^-6^ 0.52（0.40,0.69） | Reference | 5.43×10^-4^ 0.51（0.35,0.75） |
| AA/AG/GG | 62/322/437^‡^ | 27/105/89^║^ | 63/153/122^‡^ | 52/135/60^║^ |
| *P* value OR (95%CI) | Reference | 3.58×10^-4^ 1.33（1.14,1.55） | Reference | 0.01 1.29（1.06,1.57） |

^†^ Healthy control group

^║^ HBV clearance group

^‡^ HBV infection groups, including Asymptomatic HBV carriers, Chronic active hepatitis B group, HBV-related liver cirrhosis group, HBV-related heptocellular carcinoma group
