# Supplementary material for: Associations of HLA-DP Variants with Hepatitis B Virus Infection in Southern and Northern Han Chinese Populations: A Multicenter Case-Control Study
Source: PLoS One. 2011 Aug 31;6(8):e24221. doi: 10.1371/journal.pone.0024221 (PMC3164164; doi:10.1371/journal.pone.0024221)
Supplement: Table S5 — Associations of two SNPs (rs2395309, rs9277535) with HBV progression in Han Chinese populations. Compared with asymptomatic HBV group, those two sites (rs2395309 and rs9277535) in HLA-DPA1 or HLA-DPB1 gene had no associations with the chronic active hepatitis B, the HBV-related liver cirrhosis, and the HBV-related heptocellular carcinoma in southern and northern Chinese population. (DOC) [file pone.0024221.s006.doc]

**Table S5. Associations of two SNPs（rs2395309, rs9277535）with HBV progression in Han Chinese populations.**

| South of china | | | | |
| --- | --- | --- | --- | --- |
|  | AsC（n=693） | CHB（n=767） | LC（n=393） | HCC（n=336） |
| *HLA-DPB1* (Rs2395309)- dominant model(AA+AGvsGG) | | | | |
| Genotype(All subjects) |  |  |  |  |
| AA/AG/GG | 37/219/437 | 42/259/466 | 15/140/238 | 19/91/226 |
| P value  OR (95%CI) | Reference | 0.822  1.03（0.79,1.34） | 0.499  1.11（0.82,1.52） | 0.305  0.85（0.63,1.16） |
| *HLA-DPA1* (Rs9277535)- dominant model(AA+AGvsGG) | | |  |  |
| Genotype(All subjects) |  |  |  |  |
| AA/AG/GG | 60/261/378 | 60/277/433 | 26/171/196 | 31/121/188 |
| P value  OR (95%CI) | Reference | 0.501  0.92（0.71,1.18） | 0.163  1.24（0.92,1.67） | 0.881  0.98（0.73,1.31） |
| North of china | | | | |
|  | AsC（n=217） | CHB（n=197） | LC（n=151） | HCC（n=51） |
| *HLA-DPB1* (Rs2395309)- dominant model(AA+AGvsGG) | | | | |
| Genotype(All subjects) |  |  |  |  |
| AA/AG/GG | 28/92/97 | 20/84/93 | 15/57/79 | 5/16/30 |
| P value  OR (95%CI) | Reference | 0.697  0.92（0.62,1.38） | 0.189  0.74（0.48,1.16） | 0.097  0.56（0.28,1.11） |
| *HLA-DPA1* (Rs9277535)- dominant model(AA+AGvsGG) | | | | |
| Genotype(All subjects) |  |  |  |  |
| AA/AG/GG | 46/95/75 | 33/100/65 | 32/72/48 | 7/20/22 |
| P value  OR (95%CI) | Reference | 0.198  1.33（0.86,2.06） | 0.286  1.29（0.81,2.06） | 0.615  0.84（0.42,1.68） |

The *P* values, odds ratios (OR), and 95% confidence intervals (CI) were calculated on the basis of the binary logistic regression analysis, adjusted for sex and age.

Abbreviations: AsC, asymptomatic HBV carriers group; CHB,Chronic active hepatitis B group;LC,HBV-related liver cirrhosis group;HCC, HBV-related heptocellular carcinoma group; OR, odds ratio; CI, conﬁdence interval.
