# Supplementary material for: Associations of HLA-DP Variants with Hepatitis B Virus Infection in Southern and Northern Han Chinese Populations: A Multicenter Case-Control Study
Source: PLoS One. 2011 Aug 31;6(8):e24221. doi: 10.1371/journal.pone.0024221 (PMC3164164; doi:10.1371/journal.pone.0024221)
Supplement: Table S6 — A Meta-analysis for previous study and current study ( more than 2,243 cases and 4,137 controls). Genotype distributions of rs9277535 and rs2395309 in three ethnic groups (Japanese, Thais, Chinese) between healthy control group and chronic active hepatitis B group. P values of Pearson's x2 test for allele model. Odds ratios (OR) and 95% confidence intervals (CI) of minor allele from two-by-two allele frequency table. (DOC) [file pone.0024221.s007.doc]

**Table S6. Meta-analasis of the rs9277535 and rs2395309 with chronic active hepatitis B .**

| Study | Year | Population | Health | CHB | x^2^ | *P* value | OR (95%CI) |
| --- | --- | --- | --- | --- | --- | --- | --- |
|  |  |  | AA/AG/GG | AA/AG/GG |  |  |  |
| rs9277535 |  |  |  |  |  |  |  |
| Kamatani et al.  (GWAS First stage ) | 2009 | Japanese(BBJ) | 49/132/91 | 26/102/144 | 22.64 | 1.96×10^-6^ | 0.54 (0.42,0.69) |
| Kamatani et al.  (GWAS Second stage ) | 2009 | Japanese(BBJ) | 230/619/418 | 58/254/294 | 49.70 | 1.78×10^-12^ | 0.59 (0.51,0.69) |
| Kamatani et al. | 2009 | Thailander | 107/273/155 | 29/136/139 | 29.20 | 6.52×10^-8^ | 0.56 (0.46,0.69) |
| Guo et al. | 2011 | Chinese (North ) | 266/364/164 | 98/217/183 | 54.19 | 1.82×10^-13^ | 0.55 (0.47,0.65) |
| Current study |  | Chinese (South ) | 80/277/216 | 60/277/433 | 46.26 | 1.04×10^-11^ | 0.56 (0.48,0.67) |
| Current study |  | Chinese (North ) | 97/203/80 | 32/100/65 | 11.28 | 7.85×10^-4^ | 0.65 (0.51,0.83) |
| rs2395309 |  |  |  |  |  |  |  |
| Kamatani et al.  (GWAS First stage ) | 2009 | Japanese(BBJ) | 142/437/355 | 14/67/98 | 18.30 | 1.88×10^-5^ | 0.58 (0.45,0.74) |
| Kamatani et al.  (GWAS Second stage ) | 2009 | Japanese(BBJ) | 216/679/541 | 41/240/323 | 53.46 | 2.63×10^-13^ | 0.58 (0.50,0.67) |
| Guo et al. | 2011 | Chinese (North ) | 173/371/261 | 61/209/226 | 31.36 | 2.15×10^-8^ | 0.63 (0.53,0.74) |
| Current study |  | Chinese (South ) | 57/234/288 | 42/259/466 | 20.06 | 7.50×10^-6^ | 0.67 (0.56,0.80) |
| Current study |  | Chinese (North ) | 57/193/133 | 20/84/93 | 7.89 | 4.98×10^-3^ | 0.68 (0.53,0.88) |

*P* values of Pearson’s x^2^ test for allele model. Odds ratios (OR) and 95% confidence intervals (CI)of minor allele from two-by-two allele frequency table.

Abbreviations: Health, Healthy control group; CHB, Chronic active hepatitis B group; OR, odds ratios; 95%CI, 95% confidence interval.
